# Hemodynamic Reactivity to Mental Stress in Patients With Coronary Artery Disease

**DOI:** 10.1001/jamanetworkopen.2023.38060

**Published:** 2023-10-17

**Authors:** Kasra Moazzami, Brian Cheung, Samaah Sullivan, Anish Shah, Zakaria Almuwaqqat, Ayman Alkhoder, Puja K. Mehta, Brad D. Pearce, Amit J. Shah, Afif Martini, Malik Obideen, Jonathon Nye, J. Douglas Bremner, Viola Vaccarino, Arshed A. Quyyumi

**Affiliations:** 1 Emory Clinical Cardiovascular Research Institute, Division of Cardiology, Department of Medicine, Emory University School of Medicine, Atlanta, Georgia; 2 Grady Health System, Atlanta, Georgia; 3 Department of Epidemiology, Rollins School of Public Health, Emory University, Atlanta, Georgia; 4 Atlanta Veterans Affairs Medical Center, Decatur, Georgia; 5 Department of Radiology and Imaging Sciences, Emory University School of Medicine, Atlanta, Georgia; 6 Department of Psychiatry and Behavioral Sciences, Emory University School of Medicine, Atlanta, Georgia

## Abstract

**Importance:**

The clinical significance of hemodynamic reactivity to mental stress in the population with coronary artery disease (CAD) is unclear.

**Objective:**

To investigate the association between hemodynamic reactivity to mental stress and the risk of adverse cardiovascular events in patients with stable CAD.

**Design, Setting, and Participants:**

This cohort study included individuals with stable CAD from 2 prospective studies from a university-based hospital network: the Mental Stress Ischemia Prognosis Study (MIPS) and the Myocardial Infarction and Mental Stress Study 2 (MIMS2). Participants were enrolled between June 2011 and March 2016 and followed up for a median of 6.0 (IQR, 5.6-6.0) years in MIPS and 4.6 (IQR, 3.8-5.3) years in MIMS2. Data were analyzed from December 1, 2022, to February 15, 2023.

**Exposures:**

The rate-pressure product (RPP) was calculated as the mean systolic blood pressure times the mean heart rate at rest. Rate-pressure product reactivity was calculated as the maximum RPP during a standardized mental stress test minus the RPP at rest.

**Main Outcomes and Measures:**

The primary outcome was a composite of cardiovascular death or nonfatal myocardial infarction. The secondary end point additionally included hospitalizations for heart failure.

**Results:**

From the total of 938 individuals from the pooled cohort (mean [SD] age, 60.2 [10.1] years; 611 [65.1%] men), 631 participated in MIPS and 307 in MIMS2. A total of 373 individuals (39.8%) were Black, 519 (55.3%) were White, and 46 (4.9%) were of unknown race or ethnicity. The RPP increased by a mean (SD) of 77.1% (23.1%) during mental stress (mean [SD] absolute change, 5651 [2878]). For every SD decrease in RPP reactivity with mental stress, the adjusted hazard ratios for the primary and secondary end points were 1.30 (95% CI, 1.04-1.72) and 1.30 (95% CI, 1.06-1.56), respectively, in MIPS and 1.41 (95% CI, 1.06-1.97) and 1.21 (95% CI, 1.02-1.60), respectively, in MIMS2. In the pooled sample, when RPP reactivity to mental stress was added to a model including traditional clinical risk characteristics, model discrimination for adverse events improved (increase in C statistic of 5% for the primary end point; *P* = .009).

**Conclusions and Relevance:**

In this cohort study of individuals with stable CAD, a blunted cardiovascular reactivity to mental stress was associated with adverse outcomes. Future studies are needed to assess the clinical utility of mental stress reactivity testing in this population.

## Key Points

**Question:**

Is blunted hemodynamic response to mental stress associated with adverse cardiovascular events in patients with coronary artery disease?

**Findings:**

In this cohort study of 938 participants, every SD decrease in rate-pressure product reactivity with mental stress was associated with cardiovascular death or nonfatal myocardial infarction. Lower rate-pressure product reactivity with physical exercise was not associated with an elevated risk of adverse cardiovascular events.

**Meaning:**

These findings suggest that among individuals with stable coronary artery disease, a blunted cardiovascular reactivity to mental stress is associated with adverse outcomes.

## Teaser

This cohort study examines the association of cardiovascular reactivity to acute mental stress with adverse cardiovascular events among patients with stable coronary artery disease.

## Introduction

Psychological stress is associated with the development and progression of cardiovascular disease.^1^ Laboratory-based evaluation of cardiovascular reactivity to a mental stress challenge has been widely adopted as a strategy to help understand stress response physiology that may be detrimental for cardiovascular health. A number of prospective studies have shown that among people without a history of cardiovascular disease, a greater blood pressure and heart rate reactivity to mental stress is associated with an increased risk for the development of hypertension and coronary artery disease (CAD)^1,2^ and mortality due to cardiovascular disease.^3^ Given the emphasis placed on the role of greater reactivity to mental stress in worsening cardiovascular health, low or blunted reactivity to stress has, by implication, been presumed to be ultimately benign or even protective.^4,5^ However, recent evidence suggests that low cardiovascular reactivity to mental stress is associated with a number of cardiovascular risk factors such as obesity, smoking, alcohol and substance abuse, and depression.^6,7,8,9,10,11,12^

While cardiovascular reactivity to mental stress has been extensively evaluated in predominantly healthy populations, fewer and inconsistent data are available among those with stable CAD. As this population is at high risk for adverse events, it is necessary to identify factors that may contribute to the increased risk of future events in this population. Previous studies have also shown that patients with CAD are more susceptible to effects of mental stress than the general population.^1^ Although earlier studies^13,14^ have shown a positive association between greater reactivity to mental stress and worse outcomes in individuals with cardiovascular disease, more recent studies with larger sample sizes^15,16,17^ have shown that a blunted cardiovascular response to mental stress was associated with higher risk for adverse outcomes in those with recent coronary bypass grafting or with heart failure and reduced ejection fraction. Moreover, these studies have not assessed whether hemodynamic changes observed during physical stress have similar associations as changes observed during mental stress.

This study was conducted to examine the prognostic value of cardiovascular reactivity to acute mental stress in 2 parallel modern cohorts (a discovery and a validation cohort) of patients with stable CAD. We hypothesized that a blunted hemodynamic response to mental stress would be associated with worse adverse cardiovascular outcomes and that it would provide additional prognostic information compared with traditional risk models.

## Methods

### Study Participants and Protocol Overview

The research protocol for both study cohorts was approved by the Institutional Review Board of Emory University, Atlanta, Georgia, and all participants provided written informed consent. This study followed the Strengthening the Reporting of Observational Studies in Epidemiology (STROBE) reporting guideline for cohort studies, ensuring transparency, comprehensiveness, and methodological rigor in presenting findings.

Between June 2011 and March 2016, individuals with stable CAD were enrolled in 2 parallel studies with similar protocols, the Mental Stress Ischemia Prognosis Study (MIPS)^18^ and the Myocardial Infarction and Mental Stress Study 2 (MIMS2).^19^ In both cohorts, patients with stable CAD were recruited from hospitals and clinics affiliated with Emory University and shared protocols, staff, facilities, and equipment. For the MIPS cohort, participants were included if they were 30 to 79 years of age and had a documented history of CAD. For the MIMS2 cohort, inclusion criteria included a verified myocardial infarction within the past 8 months and 18 to 60 years of age at the time of the myocardial infarction. MIMS2 also included 50% women by design. Patients were excluded from both studies if they were pregnant or had medical comorbidities expected to shorten life expectancy.

At the baseline visit, each participant underwent clinical and psychosocial assessments, a standardized mental stress test, and a conventional stress test (exercise or pharmacological stress). Mental and physical or pharmacological stress testing was performed on 2 separate days up to 1 week apart. On the conventional stress testing day, participants underwent a standard Bruce protocol^20^ or, if unable to exercise, a pharmacological stress test with regadenoson (Astellas).

### Mental Stress Procedure

All patients underwent mental stress testing in the morning after a 12-hour fast. After a 30-minute rest period in a quiet room, mental stress testing was performed. Mental stress was induced by a standardized public speaking task as previously described.^18^ Each patient was given 2 minutes to prepare a speech and 3 minutes to deliver it in front of an evaluative audience of at least 4 people wearing laboratory coats. Subjective ratings of distress were obtained at rest and during mental stress testing using the Subjective Units of Distress Scale.^21^

We conducted continuous blood pressure and heart rate monitoring during the resting stage (every 5 minutes) and during the entire mental stress testing, including both preparation and speaking, and during the physical stress test (every 1 minute). We calculated the rate-pressure product (RPP) as the mean systolic blood pressure times the mean heart rate. Rate-pressure product reactivity was calculated as the maximum RPP during stress minus the minimum RPP at rest.

### Other Measures

Information on demographics (age, sex, and race and ethnicity), clinical factors (smoking, body mass index, history of hypertension, diabetes, heart failure, and previous myocardial infarction), and medications (β-blockers, statins, angiotensin-converting enzyme inhibitors, and aspirin) was obtained using standardized questionnaires and medical record reviews. Self-reported race and ethnicity data (including Black, White, and unknown race or ethnicity) were collected through questionnaires. Depressive symptoms were assessed with the Beck Depression Inventory II,^22^ general stress with the 10-item Cohen Perceived Stress Scale,^23^ and state anxiety with the Spielberger State-Trait Anxiety Inventory.^24^ An intravenous access was established for each patient and blood samples were taken at rest and at 2 minutes into the speech task to measure plasma levels of epinephrine (enzyme immunoassay kit, 2-category enzyme-linked immunosorbent assay; Labor Diagnostika Nord GmbH as supplied by Rocky Mountain Scientific Laboratory), as previously described.^25^

### Statistical Analysis

Data were analyzed from December 1, 2022, to February 15, 2023. Continuous variables are presented as mean (SD) or median (IQR), and categorical variables are presented as proportions. Linear regression models were used to examine the association between RPP reactivity with mental stress and sociodemographic factors, medical history factors, medication use, and factors associated with physical stress test. The association between RPP reactivity and adverse events was examined using Kaplan-Meier curves and Cox proportional hazards regression models. The Fine and Gray subdistribution hazard models were constructed to assess the association between RPP reactivity to mental stress and the study end points while treating noncardiovascular death as competing risk.^26^ A low or blunted RPP reactivity to stress was defined as any value less than the median. Subgroup analyses were also performed to examine whether the association of RPP reactivity to mental stress with outcomes varied according to a priori selected strata, including age (≤60 vs >60 years), sex, race and ethnicity (Black compared with White or unknown race or ethnicity), previous myocardial infarction, history of revascularization, history of heart failure, and mental stress–induced ischemia by adding the corresponding interactions in the models. We tested the incremental value of adding the RPP reactivity with mental stress to a model that included sociodemographics, traditional risk factors, current medications, and psychological factors. Significance testing was 2-sided with a significance threshold of *P* < .05, and all statistical analyses were performed using Stata software, version 14.0 (StataCorp LLC). A more detailed statistical approach is provided in the eMethods in Supplement 1.

## Results

### Study Population

From the total of 938 individuals in the pooled cohort (mean [SD] age, 60.2 [10.1] years; 611 men [65.1%] and 327 women [34.9%]), 631 participated in MIPS and 307 in MIMS2. A total of 373 individuals (39.8%) were Black, 519 (55.3%) were White, and 46 (4.9%) were of unknown race or ethnicity. Demographic and clinical characteristics of the MIPS and MIMS2 cohorts are provided in Table 1. Of the 638 patients in the MIPS, 2 withdrew before completion of baseline assessments, and 5 were lost to follow-up, leaving 631 patients for analysis. Of the 313 patients in MIMS2, 6 individuals were lost to follow-up, resulting in 307 patients for analysis.

**Table 1.** Baseline Characteristics of the 2 Study Cohorts

Characteristic	Study cohort^a^		*P* value
	MIPS (n = 631)	MIMS2 (n = 307)	
Demographic			
Age, mean (SD), y	63.9 (8.7)	50.8 (6.7)	<.001
Sex			
Men	460 (72.9)	151 (49.2)	<.001
Women	171 (27.1)	156 (50.8)	<.001
Race and ethnicity			
Black or African American	174 (27.6)	199 (64.8)	<.001
White	429 (68.0)	90 (29.3)	<.001
Unknown	28 (4.4)	18 (5.9)	.21
College education	450 (71.3)	173 (56.4)	.01
Cardiovascular risk factors			
BMI, mean (SD)	29.6 (5.3)	31.3 (7.4)	<.001
Diabetes	205 (32.5)	94 (30.6)	.76
Dyslipidemia	523 (82.9)	239 (77.9)	.32
Hypertension	473 (75.0)	244 (79.5)	.04
Smoking status			
Ever smoker	376 (59.6)	167 (54.4)	.14
Current smoker	302 (47.9)	71 (23.1)	<.001
Clinical			
Coronary artery revascularization	326 (51.7)	242 (78.8)	<.001
LVEF, mean (SD), %	66.8 (14.3)	50.7 (12.1)	<.001
History of myocardial infarction	202 (32.0)	307 (100)	<.001
History of heart failure	91 (14.4)	31 (10.1)	.08
Medications			
Aspirin	545 (86.4)	245 (79.8)	.06
Statin	540 (85.6)	256 (83.4)	.69
ACE inhibitor	284 (45.0)	141 (45.9)	.67
β-Blocker	465 (73.7)	257 (83.7)	<.001
Clopidogrel	200 (31.7)	211 (68.7)	<.001
Antidepressants	158 (25.0)	53 (17.3)	.01
Psychological factors, mean (SD)			
Beck Depression Inventory II score^b^	8.2 (8.3)	12.5 (10.7)	<.001
Cohen Perceived Stress Scale score^c^	12.1 (7.5)	16.7 (8.6)	<.001
State Anxiety score^d^	30.9 (11.0)	36.2 (13.2)	<.001

Abbreviations: ACE, angiotensin-converting enzyme; BMI, body mass index (calculated as weight in kilograms divided by height in meters squared); LVEF, left ventricular ejection fraction; MIMS2, Myocardial Infarction and Mental Stress Study 2; MIPS, Mental Stress Ischemia Prognosis Study.

^a^ Unless otherwise indicated, data are expressed as No. (%) of patients. Percentages have been rounded and may not total 100.

^b^ Scores range from 0 to 63, with higher scores indicating more severe depression.

^c^ Scores range from 0 to 40, with higher scores indicating higher perceived stress.

^d^ Measured with the Spielberger State-Trait Anxiety Inventory. Scores range from 20 to 80, with higher scores indicating greater anxiety.

### Association Between RPP Reactivity With Mental Stress and Baseline Characteristics

Results of mental and physical stress testing are given in Table 2. The RPP increased by a mean (SD) of 77.1% (23.1%) during mental stress (mean absolute change, 5651 [2878]). On multivariable linear regression analyses, a lower RPP reactivity to stress remained associated with a higher body mass index, history of smoking, use of antidepressants, lower RPP at rest, and a decrease in subjective ratings of distress and epinephrine levels with stress (eTable 1 in Supplement 1). There was no association between RPP reactivity to mental stress and any of the indices of cardiac reactivity to exercise (eTable 1 in Supplement 1). Also, a higher RPP reactivity to stress was independently associated with the presence of mental stress–induced ischemia, but not with physical stress–induced ischemia (eTable 1 in Supplement 1).

**Table 2.** Mental and Physical Stress Testing Results of the 2 Study Cohorts^a^

Test component	Study cohort		*P* value
	MIPS (n = 631)	MIMS2 (n = 307)	
Mental stress testing			
Blood pressure change, mm Hg			
Systolic	41.0 (17.8)	40.5 (16.3)	.54
Diastolic	24.3 (10.6)	28.6 (11.5)	<.001
Heart rate change, beats/min	17.0 (10.8)	23.2 (14.6)	<.001
RPP change, per 1000	5.4 (2.8)	6.2 (3.0)	<.001
Subjective Units of Distress Scale score change	9.0 (18.8)	25.5 (30.6)	<.001
Epinephrine level change, median (IQR), pg/mL	12.9 (1.8-34.3)	10.6 (3.2-23.7)	.15
Mental stress-induced ischemia, No. (%)	96 (15.2)	53 (17.3)	.44
Physical stress testing^b^			
Blood pressure change, mm Hg			
Systolic	35.8 (24.3)	54.0 (21.4)	<.001
Diastolic	13.8 (8.2)	12.3 (13.1)	.25
Heart rate change, beats/min	69.4 (17.3)	85.7 (14.1)	<.001
RPP change, per 1000	13.0 (5.7)	18.1 (3.9)	<.001
Physical stress-induced ischemia, No. (%)	211 (43.9)	77 (36.7)	<.001

Abbreviations: MIMS2, Myocardial Infarction and Mental Stress Study 2; MIPS, Mental Stress Ischemia Prognosis Study; RPP, rate-pressure product.

SI conversion factor: To convert epinephrine to mg/L, multiple by 10^−6^.

^a^ Unless otherwise indicated, data are expressed as mean (SD).

^b^ Data were available for 481 participants in MIPS and 210 participants in MIMS2.

### Association Between RPP Reactivity and Adverse Cardiovascular Outcomes

Participants were followed up for a median of 6.0 (IQR, 5.6-6.0) years in MIPS and 4.6 (IQR, 3.8-5.3) years in MIMS2. The primary end point of incident cardiovascular death or nonfatal myocardial infarction occurred in 114 of 638 individuals (17.9%) in MIPS and 50 of 313 (16.0%) in MIMS2. Corresponding numbers for the secondary end point that also included heart failure hospitalizations were 144 of 638 individuals (22.6%) in MIPS and 78 of 313 (24.9%) in MIMS2. In both populations, when dichotomized at the median, individuals with low (less than median) RPP reactivity with mental stress had a significantly elevated risk of future events compared with those with high RPP reactivity (at or above median) with mental stress (Figure). A low RPP reactivity was associated with increased risk of cardiovascular death or nonfatal myocardial infarction in both the MIPS (hazard ratio [HR], 1.42 [95% CI, 1.12-1.81]) and MIMS2 (HR, 1.89 [95% CI, 1.06-3.44]) cohorts. Similar findings were observed when using cutoffs of 1 and 2 SDs above and below the median RPP reactivity (eFigure in Supplement 1). When analyzed as a continuous variable, for every 1-SD lower RPP reactivity with mental stress, the unadjusted HR for the primary and secondary end points were 1.33 (95% CI, 1.09-1.72) and 1.36 (95% CI, 1.11-1.69), respectively, in MIPS and 1.42 (95% CI, 1.08-1.96) and 1.25 (95% CI, 1.06-1.58), respectively, in MIMS2. Adjustment for demographic, clinical, and psychological factors, medications, and changes in epinephrine levels with mental stress did not affect the association (adjusted HRs, 1.30 [95% CI, 1.04-1.72] and 1.30 [95% CI, 1.06-1.56], respectively, in MIPS and 1.41 [95% CI, 1.06-1.97] and 1.21 [95% CI, 1.02-1.60], respectively, in MIMS2) (eTable 2 in Supplement 1). In addition to RPP changes with mental stress, the presence of diabetes and mental stress–induced ischemia was independently associated with higher risk of adverse events, while taking statins was associated with lower risk (eTable 3 in Supplement 1).

**Figure.**
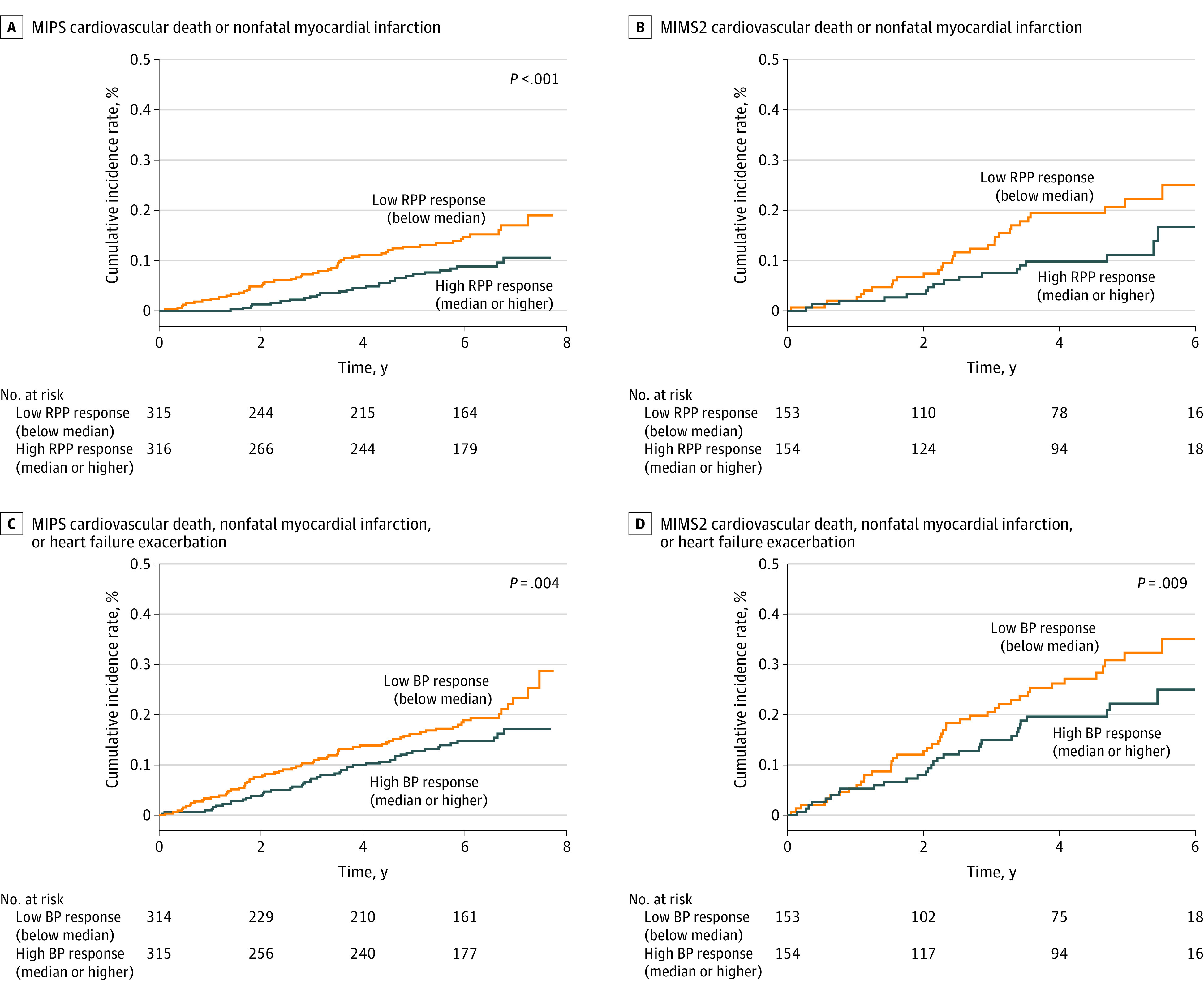
Cumulative Incidence of Cardiovascular Death, Nonfatal Myocardial Infarction, and Heart Failure Hospitalization Data are shown in the Mental Stress Ischemia Prognosis Study (MIPS) and the Myocardial Infarction and Mental Stress Study 2 (MIMS2) study populations with respect to low (less than median) and high (at or above median) rate-pressure product (RPP) reactivity to mental stress. The RPP was calculated as the mean systolic blood pressure (BP) times the mean heart rate at rest; RPP reactivity, as the maximum RPP during a standardized mental stress test minus the RPP at rest.

Similar results were found for reactivity of systolic blood pressure and heart rate with mental stress when examined separately, both for the primary and the secondary end points (Table 3). These findings were consistent in the MIPS and MIMS2 cohorts (Table 3).

**Table 3.** Association of SBP, DBP, and Heart Rate Reactivity With Mental Stress and Risk of Cardiovascular Outcomes

Outcome by study cohort	Adjusted HR (95% CI)^a^		
	SBP changes with stress	DBP changes with stress	Heart rate changes with stress
**MIPS**			
Primary^b^	1.19 (1.03-1.45)	1.02 (0.83-1.24)	1.66 (1.26-2.22)
Secondary^c^	1.26 (1.06-1.51)	1.04 (0.87-1.25)	1.69 (1.31-2.17)
**MIMS2**			
Primary^b^	1.38 (1.05-2.01)	1.17 (0.86-1.58)	1.21 (1.04-1.63)
Secondary^c^	1.21 (1.06-1.88)	1.18 (0.91-1.66)	1.17 (1.01-1.44)

Abbreviations: DBP, diastolic blood pressure; HR, hazard ratio; MIMS2, Myocardial Infarction and Mental Stress Study 2; MIPS, Mental Stress Ischemia Prognosis Study; SBP, systolic blood pressure.

^a^ Calculated for every SD reduction in SBP, DBP, or heart rate reactivity with mental stress. Adjusted for demographic factors (age, sex, and race and ethnicity), cardiovascular risk factors and other relevant medical factors (smoking, body mass index, history of hypertension, diabetes, heart failure, left ventricular ejection fraction, and previous myocardial infarction), current medications (β-blockers, statins, angiotensin-converting enzyme inhibitors, and aspirin), and psychological factors (depressive symptoms, general stress, and state anxiety and Subjective Units of Distress Scale score).

^b^ Includes cardiovascular death or nonfatal myocardial infarction.

^c^ Includes cardiovascular death, nonfatal myocardial infarction, or congestive heart failure hospitalizations.

Among the subset of participants who underwent exercise stress testing within a week from the mental stress testing, the primary and secondary end points occurred in 39 of 464 (8.4%) and 49 of 464 (10.6%), respectively, in the MIPS cohort and in 40 of 214 (18.7%) and 64 of 214 (29.9%), respectively, in the MIMS2 cohort. As shown in eTable 4 in Supplement 1, there was no association between RPP reactivity with exercise stress testing and either the primary or secondary end points.

### Risk Discrimination

Using the pooled sample, we tested the incremental value of adding the RPP reactivity with mental stress to a statistical model with baseline demographics and traditional risk factors in its association with primary and secondary end points. The C statistic for both end points increased significantly with the addition of RPP reactivity to the model: 5% for the primary end point (*P* = .009) and 6% for the secondary end point (*P* = .004) (eTable 5 in Supplement 1). The continuous net reclassification-improvement metric also showed significant reclassification of participant risk after adding RPP reactivity to mental stress: 34% for the primary end point and 41% for the secondary end point (*P* < .001 for both) (eTable 5 in Supplement 1).

## Discussion

In this pooled analysis of 2 prospective cohorts of patients with stable CAD, a blunted or lower cardiovascular reactivity to acute mental stress challenge, but not with physical exercise, was associated with an elevated risk of adverse cardiovascular events. These findings were consistently observed in each of the 2 cohorts and were independent of other clinical risk factors. Cardiovascular reactivity to mental stress was associated with modest but statistically significant improvements in the discrimination of future risk of adverse outcomes compared with a standard clinical model.

The association between cardiovascular reactivity to mental stress and future cardiovascular events in the population with CAD has been previously investigated in few studies with conflicting results.^13,14,15^ In 2 studies from the 1990s,^13,14^ a high blood pressure reactivity to mental stress was linked to adverse outcomes. However, 1 study^13^ was small, including only 13 patients following a myocardial infraction. In the other study,^14^ the authors found that individuals with higher diastolic blood pressure stress responses experienced more frequent cardiovascular events, while such associations were not found with either heart rate or systolic blood pressure changes during stress. In contrast, in a study of 521 patients who had undergone coronary bypass grafting,^15^ a low cardiovascular reactivity to mental stress was associated with a greater risk of clinical cardiovascular events. The results of our study are in line with the latter report suggesting an increased risk for clinical cardiovascular events among those with a lower reactivity to mental stress. We also did not find any associations between diastolic blood pressure responses to mental stress and future outcomes. This could partly be due to use of public speaking in our mental stress protocol, which has been shown to elucidate larger systolic blood pressure responses and smaller diastolic blood pressure responses compared with other stress protocols.^27^ Moreover, our findings showed that this effect was present irrespective of history of heart failure or revascularization.

Previous studies have also shown attenuated cardiovascular reactivity in individuals who display health behaviors associated with heightened cardiovascular disease risk. For instance, obesity, depression, smoking, and substance abuse have all been associated with a blunted cardiovascular reactivity to stress.^6,7,8,9,10,11,12^ Our study was in concordance with these results, as a lower RPP response to mental stress was associated with a higher body mass index, history of smoking, and higher burden of psychological distress. However, adjusting for these factors in the multivariable Cox proportional hazards regression models did not attenuate the association between RPP response to mental stress and future outcomes. These findings suggest that the association between lower cardiovascular reactivity and future outcomes is independent of all comorbidities and risk factors.

Our findings from the Kaplan-Meier event-free survival curves indicated that the risk associated with cardiovascular reactivity to mental stress emerged quite early in the clinical follow-up phase and persisted over time. These results, combined with the finding that reactivity to mental stress improved the C statistic and other reclassification indices when added to a standard clinical model, suggest that the hemodynamic responses to mental stress further risk stratify individuals with stable CAD beyond that provided by established risk factors.

We found an association between a higher RPP reactivity to mental stress and presence of mental stress ischemia. This association was also independent of demographic and clinical characteristics. Previous studies^28,29,30^ have also shown that ischemia with mental stress is associated with greater hemodynamic responses to stress. Our Cox proportional hazards regression models showed that both a blunted RPP reactivity to mental stress and the presence of mental stress–induced ischemia were independently associated with worse future outcomes. These findings suggest that the pathways through which hemodynamic responses to stress and ischemia with mental stress lead to worse outcomes in CAD are distinct.

In the present study, 691 participants (73.7%) underwent an exercise stress test within a week from mental stress testing. There was no association between RPP reactivity to mental stress and any of the indices of cardiac reactivity to exercise. Moreover, we did not find any association between RPP reactivity with exercise and cardiovascular outcomes. One reason for these findings could be the potential for selection bias, as the group who did not undergo exercise testing may have experienced a higher burden of comorbidities and subsequently higher risk of adverse events. However, a subgroup analysis in patients who underwent both mental and exercise stress testing showed similar findings with respect to the magnitude of association between RPP reactivity to mental stress and future outcomes. These findings suggest that the mechanisms for a blunted cardiovascular response to mental stress are different from hemodynamic responses to physical stress, and therefore cardiovascular reactivity to psychological challenge may provide unique risk stratification compared with that provided by physical assessments of functional capacity.

Why some people show blunting of cardiovascular responses to mental stress and others do not is unclear. One potential explanation is that blunted reactivity might reflect a reduced awareness or perception of stress.^31^ In other words, an inability for an individual to detect stressors in the environment could potentially dampen physiological stress responses. Our findings are in line with this theory, as we found an independent association between lower self-reported ratings of distress during the mental stress challenge and reduced RPP reactivity to mental stress. However, the addition of subjective distress ratings to our clinical model did not attenuate the association between cardiovascular reactivity to stress and future outcomes. Another potential mechanism is that these attenuated responses may reflect a reduced physiological capacity to respond to psychological stress. Our findings also support this theory, which was evidenced by a direct association between smaller changes in epinephrine levels with mental stress and lower RPP responses to mental stress. However, adjusting for changes in epinephrine levels also did not affect the association between cardiovascular reactivity to mental stress and future cardiovascular events. Therefore, while individuals with potentially diminished stress perception and/or reduced responsiveness to stressful stimuli were more likely to experience blunted stress reactivity in our study, these factors could not explain the worse cardiovascular outcomes in this group of individuals with CAD.

### Strengths and Limitations

This study is strengthened by studying 2 independent populations with CAD and producing similar results. Other strengths include a standardized mental stress protocol and concomitant exercise stress testing that enabled direct comparisons between mental and exercise stress responses.

This study also has some limitations. We used standardized mental stress testing in the laboratory setting, so its association with stress in everyday life would require further evaluation. We also only performed speaking tasks to induce mental stress and have not explored whether other types of mental stress testing could illicit similar results. Additionally, we used the maximum differences in hemodynamic responses between mental stress and resting states, rather than the mean changes with stress, which might amplify the observed effects of mental stress. Further, our study was conducted at a single institution in a population with CAD, and therefore generalizability to other populations or clinical settings requires further study.

## Conclusions

In this cohort study of individuals with stable CAD, a dampened cardiovascular reactivity to mental stress was associated with adverse outcomes and improved the discrimination of future risk of cardiovascular events beyond traditional risk indicators. Future studies are needed to assess the mechanisms behind this association and the clinical value and cost-effectiveness of testing for cardiovascular reactivity to stress in the CAD population.

## Notes

Supplement 1. **eMethods.** Statistical Approach **eTable 1.** Associations Between Rate-Pressure Product Reactivity to Mental Stress and Clinical Characteristics **eTable 2.** Association Between Rate-Pressure Product Reactivity With Mental Stress and Risk of Cardiovascular Outcomes **eTable 3.** Associations Between Clinical Characteristics and Risk of Cardiovascular Outcomes **eTable 4.** Association Between RPP Reactivity With Exercise Stress and Risk of Cardiovascular Outcomes **eTable 5.** Risk Prediction Metrics for the Rate-Pressure Product Reactivity to Mental Stress for the Primary and Secondary End Points in the Pooled Sample (n = 938) **eFigure.** Cumulative Incidence of Cardiovascular Death, Nonfatal Myocardial Infarction, and Heart Failure Hospitalization in Each of the 2 Study Populations With Respect to Cutoffs of 1 and 2 SDs Above and Below the Median RPP Reactivity Values
eReferences

Supplement 2.
Data Sharing Statement
