# Supplementary material for: Hemodynamic Reactivity to Mental Stress in Patients With Coronary Artery Disease
Source: JAMA Netw Open. 2023 Oct 17;6(10):e2338060. doi: 10.1001/jamanetworkopen.2023.38060 (PMC10582791; doi:10.1001/jamanetworkopen.2023.38060)
Supplement: Supplement 1. — eMethods. Statistical Approach eTable 1. Associations Between Rate-Pressure Product Reactivity to Mental Stress and Clinical Characteristics eTable 2. Association Between Rate-Pressure Product Reactivity With Mental Stress and Risk of Cardiovascular Outcomes eTable 3. Associations Between Clinical Characteristics and Risk of Cardiovascular Outcomes eTable 4. Association Between RPP Reactivity With Exercise Stress and Risk of Cardiovascular Outcomes eTable 5. Risk Prediction Metrics for the Rate-Pressure Product Reactivity to Mental Stress for the Primary and Secondary End Points in the Pooled Sample (n = 938) eFigure. Cumulative Incidence of Cardiovascular Death, Nonfatal Myocardial Infarction, and Heart Failure Hospitalization in Each of the 2 Study Populations With Respect to Cutoffs of 1 and 2 SDs Above and Below the Median RPP Reactivity Values eReferences [file jamanetwopen-e2338060-s001.pdf]

Supplementary Online Content

Moazzami K, Cheung B, Sullivan S, et al. Hemodynamic reactivity to mental stress in patients with coronary artery disease. *JAMA Netw Open*. 2023;6(10):e2338060. doi:10.1001/jamanetworkopen.2023.38060

eMethods. Statistical Approach

eTable 1. Associations Between Rate-Pressure Product Reactivity to Mental Stress and Clinical Characteristics

eTable 2. Association Between Rate-Pressure Product Reactivity With Mental Stress and Risk of Cardiovascular Outcomes

eTable 3. Associations Between Clinical Characteristics and Risk of Cardiovascular Outcomes

eTable 4. Association Between RPP Reactivity With Exercise Stress and Risk of Cardiovascular Outcomes

eTable 5. Risk Prediction Metrics for the Rate-Pressure Product Reactivity to Mental Stress for the Primary and Secondary End Points in the Pooled Sample (n = 938)

eFigure. Cumulative Incidence of Cardiovascular Death, Nonfatal Myocardial Infarction, and Heart Failure Hospitalization in Each of the 2 Study Populations With Respect to Cutoffs of 1 and 2 SDs Above and Below the Median RPP Reactivity Values

eReferences

This supplementary material has been provided by the authors to give readers additional information about their work.

© 2023 Moazzami K et al. *JAMA Network Open*.

eMethods. Statistical Approach

Cox proportional hazards assumptions were assessed and verified both visually by Kaplan-Meier curves and by using the linear correlation test based on Schoenfeld partial residuals of the model. All covariates also conformed with the proportionality of hazard assumption as verified by time-covariate interactions (all $P>0.05$ for interaction). Our main analysis used RPP reactivity as a continuous variable, expressed as SD decrements. Sequential models were constructed to derive hazards ratios (HRs) and 95% CIs after including prespecified variables to the unadjusted model. The fully adjusted model was used to examine the association between systolic and diastolic blood pressure and heart rate reactivity to mental stress and the primary and secondary end points. Similar sequential models were also used to examine the association between RPP changes with exercise stress testing and the primary and secondary end points. To examine consistency of results, since exercise stress testing was performed only in a portion of each cohort, a subgroup analysis was done to determine the association between RPP reactivity to mental stress and future outcomes in the subgroup who also underwent physical stress testing.

Given the similarity of results in MIPS and MIMS2 cohorts, we pooled the 2 cohorts to provide an overall estimate of effect. We used an individual patient data meta-analysis approach with random effects to preserve the clustering within each cohort.[29] Because of the potential for type I error due to multiple comparisons, findings for secondary analyses and end points should be interpreted as exploratory.

© 2023 Moazzami K et al. *JAMA Network Open*.

The Harrell C statistic (ie, area under the receiver operating characteristic curve), and category-free net reclassification improvement, were calculated as indices of risk discrimination.[30]

© 2023 Moazzami K et al. *JAMA Network Open*.

eTable 1. Associations Between Rate-Pressure Product Reactivity to Mental Stress and Clinical Characteristics

	Bivariate Analyses	Multivariable Analyses
	B (95% CI)	B (95% CI)
**Demographic Factors**		
Age, per 10 years	0.01 (-0.06, 0.05)	0.01 (-0.08, 0.04)
Male sex	**-0.19 (-0.43, -0.06)**	-0.01 (-0.1, 0.006)
Black or African American	-0.08 (-0.20, 0.04)	-0.07 (-0.18, 0.03)
**Cardiovascular Risk Factors**		
Body Mass Index	**-0.02 (-0.03, -0.009)**	**-0.02 (-0.04, -0.01)**
Diabetes	-0.04 (-0.18, 0.09)	-0.007 (-0.17, 0.16)
Dyslipidemia	-0.03 (-0.19, 0.13)	-0.02 (-0.22, 0.18)
Hypertension	0.04 (-0.11, 0.19)	0.01 (-0.18, 0.22)
Ever Smoker	**-0.12 (-0.28, -0.02)**	**-0.21 (-0.36, -0.06)**
**Clinical characteristics**		
Coronary Artery Revascularization	0.03 (-0.10, 0.16)	0.01 (-0.12, 0.11)
History of Myocardial Infarction	0.12 (-0.01, 0.25)	0.06 (-0.03, 0.38)
History of Heart Failure	**-0.29 (-0.48, -0.11)**	-0.13 (-0.37, 0.10)
Ejection Fraction	**0.005 (0.001, 0.009)**	0.004 (-0.002, 0.10)
**Medications**		
Aspirin	0.13 (-0.04, 0.31)	0.08 (-0.02, 0.37)
Statin	-0.06 (-0.24, 0.11)	-0.02 (-0.18, 0.12)
Angiotensin Converting Enzyme Inhibitor	-0.08 (-0.21, 0.04)	-0.09 (-0.23, 0.05)
Beta Blocker	-0.12 (-0.27, 0.03)	-0.09 (-0.22, 0.02)
Clopidogrel,	-0.01 (-0.13, 0.12)	-0.009 (-0.11, 0.09)
Antidepressants	**-0.31 (-0.47, -0.16)**	**-0.34 (-0.53, -0.16)**
**Mental Stress Testing**		
Resting RPP	**0.10 (0.03, 0.16)**	**0.18 (0.10, 0.26)**
Subjective Units of Distress Scale, change	**0.06 (0.02, 0.09)**	**0.05 (0.01, 0.08)**
Epinephrine, change	**0.01 (0.009, 0.013)**	**0.01 (0.008, 0.01)**
Mental Stress-Induced Ischemia	**0.29 (0.11, 0.47)**	**0.30 (0.12, 0.47)**
**Physical Stress testing**		
Blood pressure, change, mm Hg		
Systolic	0.003 (-0.01, 0.01)	0.002 (-0.01, 0.01)
Diastolic	0.008 (-0.01, 0.03)	-0.008 (-0.01, 0.02)
Heart rate, change, beats/minute	0.009 (-0.004, 0.02)	0.005 (-0.003, 0.06)
RPP, change, per 1000	0.003 (-0.004, 0.007)	0.001 (-0.005, 0.006)
Total exercise time	-0.03 (-0.07, 0.02)	-0.04 (-0.09, 0.03)
Exercise stress test	**0.25 (0.10, 0.38)**	0.07 (-0.10, 0.24)
Physical Stress-Induced Ischemia	0.01 (-0.14, 0.16)	0.02 (-0.12, 0.16)
**Psychological Factors, mean (SD)**		
Beck Depression Inventory II	**-0.01 (-0.01, -0.003)**	-0.04 (-0.15, 0.06)

Cohen Perceived Stress Scale 10	**-0.09 (-0.16, -0.03)**	0.04 (-0.03, 0.13)
State Anxiety	**-0.10 (-0.16, -0.03)**	-0.04 (-0.19, 0.01)

RPP: Rate-Pressure Product; Bold: statistical significance at P<0.05

© 2023 Moazzami K et al. *JAMA Network Open*.

eTable 2. Association Between Rate-Pressure Product Reactivity With Mental Stress and Risk of Cardiovascular Outcomes

	MIPS	MIMS2
	HR (95% CI)*	
**Cardiovascular death or nonfatal MI**		
Unadjusted	1.33 (1.09, 1.72)	1.42 (1.08, 1.96)
Adjusted for demographic factors[a]	1.31 (1.08, 1.70)	1.41 (1.07, 1.87)
Above variables + Clinical risk factors[b]	1.30 (1.06, 1.69)	1.40 (1.06, 2.00)
Above variables + Medications[c]	1.31 (1.08, 1.74)	1.40 (1.06, 2.01)
Above variables + Psychological factors[d] and Subjective Units of Distress Scale + Mental stress-induced ischemia	1.31 (1.05, 1.71)	1.40 (1.05, 1.95)
Above variables + Epinephrine changes with mental stress	1.30 (1.04, 1.72)	1.41 (1.06, 1.97)
**Cardiovascular death, nonfatal MI or CHF hospitalizations**		
Unadjusted	1.36 (1.11, 1.69)	1.25 (1.06, 1.58)
Adjusted for demographic factors[a]	1.37 (1.11, 1.71)	1.24 (1.05, 1.55)
Above variables + Clinical risk factors[b]	1.36 (1.10, 1.68)	1.24 (1.03, 1.57)
Above variables + Medications[c]	1.33 (1.09, 1.61)	1.23 (1.04, 1.56)
Above variables + Psychological factors[d] and Subjective Units of Distress Scale + Mental stress-induced ischemia	1.31 (1.06, 1.60)	1.22 (1.03, 1.59)
Above variables + Epinephrine changes with mental stress	1.30 (1.06, 1.55)	1.20 (1.01, 1.60)

*Hazard ratio calculated for every standard deviation reduction in RPP reactivity with mental stress

[a]Age, sex, and race (Black vs non-Black participants).

[b] Ever smoking, body mass index, history of hypertension, history of diabetes, history of dyslipidemia, history of heart failure, left ventricular ejection fraction, previous myocardial infarction, and resting rate-pressure product

[c] β-Blockers, statins, angiotensin-converting enzyme inhibitors, and aspirin.

[d] Depressive symptoms (Beck Depression Inventory II score), perceived stress (Cohen Perceive Stress Scale 10 score), and state anxiety (Spielberger State-Trait Anxiety Inventory).

© 2023 Moazzami K et al. *JAMA Network Open*.

eTable 3. Associations Between Clinical Characteristics and Risk of Cardiovascular Outcomes

	**HR (95% CI)**
**Demographic Factors**	
Age, per 10 years	1.01 (0.96, 1.03)
Female sex	1.01 (0.69, 1.46)
Black or African American	1.34 (0.91, 1.98)
**Cardiovascular Risk Factors**	
Body Mass Index	1.01 (0.98, 1.04)
Diabetes	**1.61 (1.12, 2.29)**
Dyslipidemia	1.29 (0.78, 2.13)
Hypertension	1.76 (1.01, 3.01)
Ever Smoker	1.24 (0.87, 1.76)
**Clinical characteristics**	
Coronary Artery Revascularization	0.68 (0.47, 1.02)
History of Myocardial Infarction	1.16 (0.77, 1.73)
History of Heart Failure	1.47 (0.95, 2.28)
Ejection Fraction	0.97 (0.95, 1.01)
**Medications**	
Aspirin	0.83 (0.55, 1.27)
Statin	**0.54 (0.33, 0.87)**
Angiotensin Converting Enzyme Inhibitor	1.11 (0.80, 1.54)
Beta Blocker	1.56 (0.92, 2.64)
Clopidogrel	1.32 (0.87, 1.96)
Antidepressants	1.20 (0.80, 1.76)
**Mental Stress Testing**	
Resting RPP, per 1000	1.17 (0.98, 1.39)
RPP Change, per 1000	**0.81 (0.67, 0.98)**
Subjective Units of Distress Scale, change	0.98 (0.96, 1.02)
Presence of Mental stress induced Ischemia	**1.58 (1.11, 2.41)**
**Physical Stress testing**	
Presence of Conventional Stress induced ischemia	0.95 (0.65, 1.39)
**Psychological Factors, mean (SD)**	
Beck Depression Inventory II	0.98 (0.96, 1.01)
Cohen Perceived Stress Scale 10	1.01 (0.97, 1.03)
State Anxiety	1.01 (0.98, 1.03)

© 2023 Moazzami K et al. *JAMA Network Open*.

eTable 4. Association Between RPP Reactivity With Exercise Stress and Risk of Cardiovascular Outcomes

	MIPS	MIMS2
	HR (95% CI)*	
**Cardiovascular death or nonfatal MI**		
Unadjusted	1.21 (0.89, 1.64)	1.09 (0.80, 1.49)
Adjusted for demographic factors[a]	1.20 (0.88, 1.62)	1.04 (0.74, 1.45)
Above variables + Clinical risk factors[b]	1.19 (0.87, 1.61)	1.01 (0.70, 1.47)
Above variables + Medications[c]	1.19 (0.86, 1.61)	1.01 (0.70, 1.44)
Above variables + Psychological factors[d] and Subjective Units of Distress Scale	1.18 (0.84, 1.59)	1.00 (0.68, 1.39)
Above variables + Epinephrine changes with mental stress	1.16 (0.80, 1.55)	1.00 (0.67, 1.38)
**Cardiovascular death, nonfatal MI or CHF hospitalizations**		
Unadjusted	1.29 (0.93, 1.66)	1.40 (0.96, 1.81)
Adjusted for demographic factors[a]	1.33 (0.96, 1.74)	1.26 (0.91, 1.63)
Above variables + Clinical risk factors[b]	1.31 (0.93, 1.72)	1.26 (0.90, 1.61)
Above variables + Medications[c]	1.29 (0.92, 1.70)	1.25 (0.89, 1.64)
Above variables + Psychological factors[d] and Subjective Units of Distress Scale	1.30 (0.88, 1.72)	1.24 (0.88, 1.59)
Above variables + Epinephrine changes with mental stress	1.28 (0.90, 1.69)	1.23 (0.88, 1.58)

*Hazard ratio calculated for every standard deviation reduction in RPP reactivity with mental stress

[a]Age, sex, and race (Black vs non-Black participants).

[b] Ever smoking, body mass index, history of hypertension, history of diabetes, history of dyslipidemia, history of heart failure, left ventricular ejection fraction, previous myocardial infarction, and resting RPP

[c] β-Blockers, statins, angiotensin-converting enzyme inhibitors, and aspirin.

[d] Depressive symptoms (Beck Depression Inventory II score), perceived stress (Cohen Perceive Stress Scale 10 score), and state anxiety (Spielberger State-Trait Anxiety Inventory

© 2023 Moazzami K et al. *JAMA Network Open*.

eTable 5. Risk Prediction Metrics for the Rate-Pressure Product Reactivity to Mental Stress for the Primary and Secondary End Points in the Pooled Sample (n = 938)

	**C-Statistics (95% CI)**	**ΔC-statistic (95% CI)**	**Continuous NRI (95% CI)**
**Primary Endpoint (Cardiovascular death or nonfatal MI)**			
**Model**	0.71 (0.61 – 0.80)	-	
**Model + RPP reactivity**	0.76 (0.68 – 0.87)	0.05 (0.01 – 0.12)	0.34 (0.17 – 0.58)
**Secondary Endpoint (Cardiovascular death, nonfatal MI or CHF hospitalizations)**			
**Model**	0.73 (0.64 – 0.86)	-	
**Model + RPP reactivity**	0.80 (0.68 – 0.87)	0.06 (0.02 – 0.14)	0.41 (0.22 – 0.71)

MI: Myocardial Infarction; CHF: Congestive Heart Failure; RPP: rate-pressure product; NRI: net reclassification index,

Model included demographic factors (age, sex, and race), cardiovascular risk factors and other relevant medical factors (smoking, body mass index, history of hypertension, diabetes, heart failure, and previous myocardial infarction), current medications (β-blockers, statins, angiotensin-converting enzyme inhibitors, and aspirin), and psychological factors (depressive symptoms, general stress, and state anxiety and Subjective Units of Distress Scale)

© 2023 Moazzami K et al. *JAMA Network Open*.

eFigure. Cumulative Incidence of Cardiovascular Death, Nonfatal Myocardial Infarction, and Heart Failure Hospitalization in Each of the 2 Study Populations With Respect to Cutoffs of 1 and 2 SDs Above and Below the Median RPP Reactivity Values

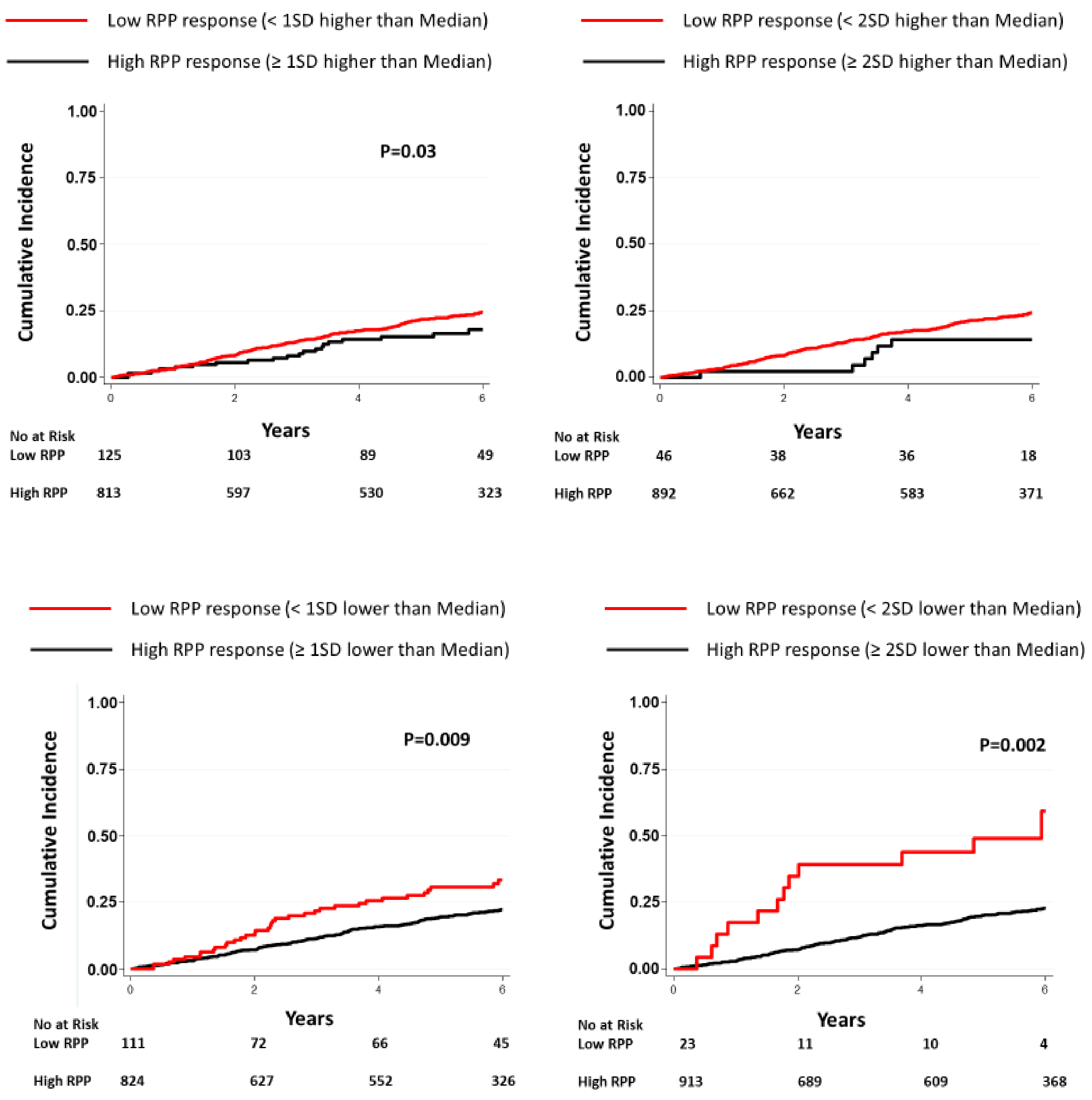

© 2023 Moazzami K et al. *JAMA Network Open*.

eReferences

1. Hammadah M, Al Mheid I, Wilmot K, et al. The Mental Stress Ischemia Prognosis Study: Objectives, Study Design, and Prevalence of Inducible Ischemia. *Psychosom Med.* 2017;79(3):311-317.
2. Vaccarino V, Sullivan S, Hammadah M, et al. Mental Stress-Induced-Myocardial Ischemia in Young Patients With Recent Myocardial Infarction: Sex Differences and Mechanisms. *Circulation.* 2018;137(8):794-805.

© 2023 Moazzami K et al. *JAMA Network Open*.
