# Supplementary material for: Hemodynamic Reactivity to Mental Stress in Patients With Coronary Artery Disease
Source: JAMA Netw Open. 2023 Oct 17;6(10):e2338060. doi: 10.1001/jamanetworkopen.2023.38060 (PMC10582791; doi:10.1001/jamanetworkopen.2023.38060)
Supplement: Supplement 2. — Data Sharing Statement [file jamanetwopen-e2338060-s002.pdf]

Data Sharing Statement

Moazzami. Hemodynamic Reactivity to Mental Stress in Patients With Coronary Artery Disease. *JAMA Netw Open*. Published October 17, 2023.
doi:10.1001/jamanetworkopen.2023.38060

Data
Data available: Yes
Data types: Deidentified participant data
How to access data: can be requested from kmoazza@emory.edu
When available: With publication

Supporting Documents
Document types: None

Additional Information
Who can access the data: researchers whose proposed use of the data has been approved
Types of analyses: for any purpose
Mechanisms of data availability: after approval of a proposal
